# Advances in natural compound-based nanomedicine and the interaction with gut microbiota in ulcerative colitis therapy

**DOI:** 10.3389/fphar.2023.1197144

**Published:** 2023-07-13

**Authors:** Jinlan Zhang, Shuhui Sun, Huan Chen, Yifan Feng, Ying Li, Zhengqi Dong

**Affiliations:** ^1^ Drug Delivery Research Center, Institute of Medicinal Plant Development, Chinese Academy of Medical Sciences & Peking Union Medical College, Beijing, China; ^2^ Key Laboratory of Bioactive Substances and Resources Utilization of Chinese Herbal Medicine, Chinese Academy of Medical Sciences & Peking Union Medical College, Beijing, China; ^3^ Key Laboratory of New Drug Discovery Based on Classic Chinese Medicine Prescription, Chinese Academy of Medical Sciences, Beijing, China; ^4^ Beijing Key Laboratory of Innovative Drug Discovery of Traditional Chinese Medicine (Natural Medicine) and Translational Medicine, Chinese Academy of Medical Sciences & Peking Union Medical College, Beijing, China

**Keywords:** ulcerative colitis, natural compounds, gut microbiota, nanomedicines, interactions

## Abstract

Ulcerative colitis (UC) is a chronic inflammatory bowel disorder of the large intestine. Previous studies have indicated that the gut microbiota plays an important role in the triggers, development, and treatment response of UC. Natural active molecules and their nanoformulations show huge potential for treating UC. The nanoparticles can regulate the gut microbiota and metabolites, whereas gut microbiota-mediated effects on nanomedicines can also bring additional therapeutic benefits. Therefore, this review aims to integrate current research on natural active molecule-based nanomedicines for UC therapy and their interaction with the gut microbiota. Here, this discussion focuses on the effects and functions of gut microbiota and metabolites in UC. The use of active molecules and the nanoformulation from natural compounds for UC therapy have been provided. The interactions between the gut microbiota and nanomedicines are derived from natural products and elucidate the possible biological mechanisms involved. Finally, the challenges and future directions for enhancing the therapeutic efficacy of nanomedicine in treating UC are proposed.

## 1 Introduction

Inflammatory bowel disease (IBD) is a group of chronic inflammatory disorders of the gastrointestinal tract with high recurrence and a long course. Ulcerative colitis (UC) is the most common large intestine IBD that causes mucosal inflammation and a dysregulated immune response, resulting in bloody stool and diarrhea (Gajendran et al., 2019). The incidence of UC has increased, and it has become a major worldwide public health concern. The highest incidences of UC have been reported in Northern Europe (24.3 per 100,000) and Canada (19.2 per 100,000). Moreover, its increasing prevalence among the young population is concerning (Ng et al., 2017). UC, being a chronic condition that lasts a lifetime, imposes substantial costs and creates a global healthcare burden (Du and Ha, 2020).

The drug treatments for UC (aminosalicylic acid, immunomodulators, corticosteroids, and monoclonal antibodies) provide only temporary relief with side effects and drug resistance (Bischoff et al., 2023). Therefore, there is an urgent need to identify better therapeutics, especially those targeted in the inflamed colon with low side effects. Natural product molecules have been reported to be effective in treating UC based on high anti-inflammatory effects and well-known safety. The presence of nano-delivery systems increases the effectiveness of natural bioactive molecules by enhancing their stability and solubility and providing a platform for their delivery to the inflamed part of the intestine (Zhang and Merlin, 2018).

Uncertainty surrounds the pathogenesis of UC, which may be related to the host genes, environment, immune dysregulation, and gut microbiota (Kudelka et al., 2020). Early studies reveal that the symbiotic microorganisms living in the intestinal tract play a crucial role in the triggers, development, and treatment response of UC. In UC, the gut microbiota exhibited reduced abundance and diversity, specific taxa changes, and altered microbial metabolites (Caruso et al., 2020). Natural products show great therapeutic potential against UC. The review has focused on using natural products and nanoformulation in treating UC. Due to their high stability and bioavailability, nanoparticles offer a promising UC treatment platform (Zu et al., 2021). Nanoparticle–microbiota interactions should be considered when using nanoparticles in UC treatment to improve UC therapy.

Alterations in the gut microbiome associated with UC are discussed in this review, as well as the use of natural products and nanomedicines in treating UC. Finally, the recent progress in nanomedicines based on natural products for interactions with the gut microbiota is being investigated as a potential new therapeutic approach against UC. This work could potentially promote the development and application of nanotechnology in intestinal diseases.

## 2 The alteration of the gut microbiota and metabolites in UC

The gut microbiota plays critical physiological roles in host metabolism and metabolic disorders. Previous studies have indicated that there is an association between the dysfunction of the gut microbiota and the occurrence and aggravation of UC (Table 1). The changes in the intestinal microbiota could lead to the fluctuation of the composition and concentration of metabolites (Rooks and Garrett, 2016).

**TABLE 1:** Alteration of the gut microbiota and/or metabolites in UC in the past 10 years.

Sample type	Sample size	Method	Major altered microbiota and/or the metabolites	Reference
Colonoscopic biopsy specimens	33 patients with UC and 18 healthy controls	Quantitative real-time PCR	Enterobacteria, *Desulfovibrio*, type E *Clostridium perfringens*, and *Enterococcus faecalis*↑; and *Clostridium butyricum*, *Ruminococcus albus*, *Eubacterium rectale*, *Lactobacillus* and *Bifidobacterium*↓	Fite et al. (2013)
Fecal samples	127 patients with UC and 87 healthy controls	PCR-DGGE and real-time PCR/gas chromatography–mass spectrometry	*Roseburia hominis* and *Faecalibacterium prausnitzii*↓, and propionic acid and acetic acid↓	Machiels et al. (2014)
Fecal samples	44 patients with UC and 21 healthy controls	1H nuclear magnetic resonance (NMR) spectroscopy	Butyrate and propionate↓	Bjerrum et al. (2015)
Luminal, mucosal, and mucus gel samples	five patients with UC and four healthy controls	16S rRNA profiling targeting the V4 region	Bacteroidaceae and Akkermansiaceae ↓	Lavelle et al. (2015)
			Clostridiaceae, Peptostreptococcaceae, Enterobacteriaceae, Ruminococcaceae, Bifidobacteriaceae, Actinomycetaceae, and an uncultured member of the Prevotellaceae family↑	
Fecal samples	30 patients with UC and 13 healthy controls	16S rRNA profiling targeting the V4 region	α-diversity↓; specific *Bacteroides*, Prevotella species, and unclassified members of the families Lachnospiraceae and Ruminococcaceae↓; and	Mar et al. (2016)
			*Streptococcus*, *Bifidobacterium*, and *Enterococcus* genera ↑	
Fecal samples	six patients with UC and six healthy controls	Metagenomic sequencing	Species richness and diversity↓	Knoll et al. (2017)
			*Eubacterium rectale* and *Faecalibacterium prausnitzii*↓	
			*Escherichia coli*↑	
Fecal samples	82 patients with UC and 51 healthy controls	LC-QTOF-MS analysis	Biogenic amines (putrescine and cadaverine)↑	Santoru et al. (2017)
Fecal samples	27 patients with UC and 25 healthy controls	hsp60-based microbiome analysis	Bacteroidetes OTU number and species diversity↓	Ishikawa et al. (2018)
			Bacteroidaceae↓	
			Porphyromonadaceae↓	
			Prevotellaceae↑	
			Sphingobacteriales↑	
Fecal samples	51 patients with UC and 73 healthy controls	Bar-coded 16S rRNA amplicon sequencing	Alpha diversity↓	Zhou et al. (2018)
			Bacteroidetes (Bacteroidia)↑	
			Pseudomonadaceae↑	
			*Streptococcus*↑	
			Proteobacteria phylum and Bacilli class ↑	
			Clostridiales↓	
Colonoscopic biopsy specimens	40 patients with UC and 40 healthy controls	16S rRNA with gene-targeted and species-specific amplicon sequencing	*Faecalibacterium prausnitzii*↓	Al-Bayati et al. (2018)
			Prevotella↓	
			*Peptostreptococcus*↓	
Colonoscopic biopsy specimens	26 patients with UC and 27 healthy controls	High-throughput 16S rDNA sequencing	Bacteroidetes↓	Jalanka et al. (2020)
			Peptostreptococcaceae and Enterobacteriaceae↑	
Colonoscopic biopsy specimens	80 patients with UC and 31 healthy controls	16S rRNA V3–V4 amplicon sequencing	Microbiota diversities↓, *Anaerostipes hadrus*, and an unclassified species of the Lachnospiraceae↓	Ryan et al. (2020)
			*Gemmiger formicilis*↓	
			*Bacteroides dorei* and *Bacteroides vulgatus*↑	
Fecal samples	10 patients with UC and 10 healthy controls	16S rRNA gene sequencing	Microbiome diversity↓	Zakerska-Banaszak et al. (2021)
			Proteobacteria, actinobacteria, and candidate division TM7 ↑	
			Bacteroidetes and Verrucomicrobia↓	
Fecal samples	13 patients with UC and 48 healthy controls	Shotgun metagenomics	Alpha diversity↓	Galipeau et al. (2021)
			*Adlercreutzia equolifaciens*↓	
			*Bilophilas*↓	
			*Bifidobacterium*↓	
Colonoscopic biopsy specimens	52 patients with UC and 34 healthy controls	HSP60 as a target in bacterial metagenome analysis	*Alistipes putredinis*, *Bacteroides coprocola*, *Bacteroides uniformis*, *Bacteroides cellulosilyticus*, *Bacteroides intestinalis*, and *Parabacteroides goldsteinii*↓	Nomura et al. (2021)
Fecal samples	32 patients with UC and 23 healthy controls	UPLC-MS	Deoxycholic acid and lithocholic acid↓	Yang et al. (2021)
Fecal samples	46 patients with UC and 36 healthy controls	16S rRNA V3–V4 amplicon sequencing	Alpha diversity↓, and *Bifidobacterium adolescentis* and *Haemophilus parainfluenzae* ↑	Barberio et al. (2022)
			*Akkermansia muciniphila* and *Coprococcus eutactus*↓	
Fecal samples	4 patients with UC and 10 healthy controls	16S rRNA metagenomic sequencing	*Faecalibacterium prausnitzii* and *Prevotella sp*.↓	Dahal et al. (2023)
			*Ligilactobacillus ruminis*↑	
			*Enterococcus faecium*, *Enterococcus faecalis*, and *Escherichia coli*↑	

### 2.1 The normal gut microbiota

The gut microbiota is a complex community of microorganisms that reside in the gastrointestinal tract and consist of thousands of microorganisms, including bacteria, viruses, and a few eukaryotes (Passos and Moraes-Filho, 2017). Two major phyla, namely, Bacteroidetes and Firmicutes, followed by Proteobacteria, Fusobacteria, Tenericutes, Actinobacteria, and Verrucomicrobia, constituted 90% of the total microbial population in the normal human gut microbiota (Jethwani and Grover, 2019). Bacteroidetes include *Bacteroides, Prevotella, Parabacteroides,* and *Alistipes*. Firmicutes consist of *Faecalibacterium*, *Clostridium*, *Eubacterium*, *Roseburia*, *Blautia*, *Lactobacillus*, and *Ruminococcus* (Miquel et al., 2015; Mukherjee et al., 2020). In normal conditions, the gut microbiota interacts with the host and plays beneficial roles for the host. The gut environment promotes microbial community growth, reproduction, and longevity (Browne et al., 2016). Maintaining a healthy gut microbiota is essential for a symbiotic relationship with the host. However, there is no “gold standard” reference to the human gut microbiota capable of promoting host metabolic health.

### 2.2 The connections between the gut microbiota and UC

Multiple studies have shown that UC patients have decreased intestinal microbial diversity and abundance, an increase in the pro-inflammatory bacteria community, a decrease in the anti-inflammatory bacteria community (Burrello et al., 2018), and an expansion and penetration of pathogens (Guo et al., 2020). Firmicutes are related to the protective function of the intestinal barrier. The gut microbiota is characterized by low diversity and abundance of Firmicutes in UC patients. *Roseburia hominis* and *Faecalibacterium prausnitzii* decreased in UC patients compared to the controls (Machiels et al., 2014; Knoll et al., 2017; Dahal et al., 2023). *F. prausnitzii*, a member of the *Clostridium leptum* cluster, promotes intestinal health by stimulating the production of regulatory T cells and anti-inflammatory cytokines, thereby exhibiting a protective effect (Ohkusa and Koido, 2015). A decrease in the abundance of *Eubacterium rectale, Clostridium butyricum*, *Ruminococcus albus*, *Lactobacillus*, and some unclassified members of the Lachnospiraceae families was found in UC patients (Fite et al., 2013; Mar et al., 2016; Ryan et al., 2020). *Bacteroides* spp. inhibit the outer mucosal layer of the colon and have significant functions in the digestion of complex carbohydrates. *Bacteroides*, including *B. coprocola*, *B. uniformis*, *B. cellulosilyticus*, and *B. intestinalis*, were significantly reduced in UC patients, especially during an active phase (Ishikawa et al., 2018; Nomura et al., 2021). Polysaccharide A, expressed on the surface of *Bacteroides fragilis*, promotes regulatory T-cell growth and cytokine expression, protecting UC (Zhou and Zhi, 2016). However, *B. vulgatus* disrupts colonic epithelial integrity correlated with the onset of UC caused by increased serine or cysteine proteases (Mills et al., 2022).

In clinical trials, an imbalance in intestinal flora in UC patients is accompanied by an elevated abundance of Proteobacteria. For instance, Enterobacteriaceae and Desulfovibrionaceae are potent stimulators of inflammation (Shin et al., 2015). Known pathobionts, such as *Escherichia coli,* were enriched in UC patients (Knoll et al., 2017). In addition, Lavelle et al. demonstrated changes in mucolytic bacteria in UC patients. *Akkermansia muciniphila*, a species that utilizes colonic mucin as its substrate, was found to be reduced (Lavelle et al., 2015). Studies on humans and mice have shown that probiotics, specifically *Lactobacillus* and *Bifidobacterium*, may be a promising administration option for UC (Chibbar and Dieleman, 2015). The genus *Adlercreutzia* was decreased in UC. *Adlercreutzia* can affect the metabolism of phenolic compounds, thus reducing the antibacterial and anti-inflammatory effects of phenols (Galipeau et al., 2021). There are reports of a decrease in *Verrucomicrobia, Roseburia,* and *Akkermansia*, and an increase in potential pathogens containing *Streptococcus* and *Enterococcus* in UC patients (McIlroy et al., 2018). Disorders of the intestinal flora are closely related to UC. However, the specific bacteria related to the development of UC remain unknown. According to the available evidence, structural and diversity changes in the gut microbiota may play a crucial role in UC progression.

### 2.3 The metabolites of the gut microbiota associated with UC

Certain microbial-derived metabolite classes, specifically short-chain fatty acids (SCFAs), bile acids, and indole compounds, are associated with the pathogenesis and progression of UC. In UC patients, SCFAs have a negative correlation with disease progression. Bjerrum et al. found that SCFA (propionic acid and butyric acid) contents of the intestines in UC patients were decreased (Bjerrum et al., 2015). Machiels et al. reported that propionic acid and acetic acid decreased in the fecal matter of UC patients (Machiels et al., 2014). SCFAs, produced from fermentable non-digestible carbohydrates, serve as an energy source for the host and demonstrate anti-inflammatory effects (Parada Venegas et al., 2019). Butyrate is the preferred energy substrate for colonocytes. The molecular mechanisms involved in the anti-inflammatory effects are due to NF-κB and interferon-γ signaling inhibitor activity. *F. prausnitzii* is one of the most abundant butyrate-producing species (Lenoir et al., 2020). Li et al. showed that butyric acid and propionic acid could affect the secretion of inflammatory factors, inhibiting IL-8 and increasing IL-33 (Li et al., 2021). Acetate promotes mucosal homeostasis by inducing T-cell-dependent IgA production and modulating commensal bacterial composition (Takeuchi et al., 2021). During intestinal growth, *A. muciniphila*, the mucin-degrading bacteria, can produce acetate and propionate (Bian et al., 2019). Current knowledge supports that SCFAs can also regulate the expression of genes involved in energy metabolism, promote the proliferation of epithelial cells, and maintain the colonic epithelial barrier (Hosseinkhani et al., 2021).

The bile acids are the final metabolites of cholesterol in the liver. Specific gut microbes convert primary bile acids into secondary bile acids (SBAs) once they reach the colon. Yang et al. showed that the concentration of SBAs (deoxycholic acid and lithocholic acid) in the feces of UC patients was significantly lower than that of controls and positively associated with *Roseburia, Clostridium IV, Butyricicoccus*, and *Faecalibacterium* (Yang et al., 2021). Lajczak-McGinley et al. showed that SBAs could relieve inflammation by reducing the production of pro-inflammatory factors and inhibiting the apoptosis of intestinal epithelial cells (Lajczak-McGinley et al., 2020).

Indole compounds are ligands for endogenous aromatic receptors (AhR), which are the products of tryptophan metabolism by *Lactobacillus* and *Allobaculum*. Stimulation of the AhR signaling pathway increases IL-22 secretion and tight junction protein production, suggesting that indole compounds can protect the intestinal mucosal barrier (Sugimoto et al., 2016). After inoculating DSS mice with three *Lactobacillus* strains with high tryptophan metabolic activity, the AhR ligands were upregulated, and there was an increase in anti-inflammatory cytokine (IL-10) production and tight junction proteins (Shi et al., 2020).

The metabolism of indigestible nitrogen components in the diet can produce bioamines. An increase in intestinal inflammatory infiltration is associated with UC progression, and amines in the colon are derived mainly from the metabolism of indigestion-related proteins by intestinal flora. In a clinical study, fecal samples were analyzed using metabolomics and metagenomics. Putrescine and cadaverine were significantly higher in UC patients than in healthy people, suggesting putrescine and cadaverine may promote the occurrence of UC (Santoru et al., 2017). In an *in vitro* culture of Caco-2 and T84 cells, putrescine was observed to disrupt epithelial tight junctions, and putrescine intervention increased colon inflammation in DSS mice (Ghosh et al., 2021).

## 3 The well-known natural products and their nanoformulation for UC treatment

Natural products (NPs), specifically those derived from plants, are chemical molecules with abundant biological or pharmacological activities. Due to their high activity, structural specificity, and diverse therapeutic mechanisms, NPs have gradually become an essential source of anti-inflammatory drugs for treating UC (Table 2) (Cao et al., 2022). However, in actual application, most NPs are restricted by poor solubility, stability, solubility, and lack of targeting specificity (Rajendran et al., 2021). Due to the solubility and stability of the NPs, which target the delivery of NPs to the colon, nano-drug delivery systems show great potential.

**TABLE 2:** Well-known natural products and nanoformulation with a protective effect in UC.

Natural product/nano formulation	Modeling	Therapeutic effects	Reference
Kaempferol	DSS-induced colitis in C57BL/6J mice	Modulates inflammatory biomarkers and protects colonic mucosa	Park et al. (2012)
Quercetin	Caco-2 cells	Enhances the expression of zona occludens (ZO)-2, occludin, and claudin-4	Suzuki and Hara (2009)
Isorhamnetin	DSS-induced colitis in C57BL/6J mice	Promotes MPO activity and inhibits TNF-α and IL-6 levels	Dou et al. (2014)
Epigallocatechin gallate (EGCG)	DSS-induced colitis in C57BL/6J mice	Enriches short-chain fatty acid (SCFA)-producing bacteria	Wu et al. (2021)
Curcumin	Patients with UC	Prevents the relapse of UC	Lang et al. (2015)
Resveratrol	Patients with UC	Reduces oxidative stress and inflammation	Samsami-Kor et al. (2015)
Juglone	DSS-induced colitis in ICR mice	Regulates intestinal flora and Th17/Treg homeostasis	Hua et al. (2021)
Tanshinone IIa	DSS-induced colitis in C57BL/6J mice	Regulates taurine and hypotaurine metabolism	Zhu et al. (2022)
Geraniol	DSS-induced colitis in C57BL/6J mice	Reduces pro-inflammatory cytokine content and myeloperoxidase activity, and restores the decreased antioxidant parameters	Medicherla et al. (2015)
Cyclocitane-type triterpenoids	DSS-induced colitis in C57BL/6J mice	Inhibit cyclooxygenase, 5-lipoxygenase, and protein denaturation	Marius et al. (2020)
Oxymatrine	DSS-induced colitis in BALB/c mice	Promotes anti-inflammatory and pro-apoptotic activities, and downregulates the differentiation of Th1 and Th17 cells	Chen et al. (2017)
Berberrubine	DSS-induced colitis in C57BL/6J mice	Recognizes bitter taste receptors on intestinal tuft cells and promotes the differentiation of intestinal stem cells	Xiong et al. (2021)
Pectic polysaccharides AL-I from *Aconitum carmichaelii* leaves	DSS-induced colitis in C57BL/6 mice	Alleviate the symptoms, improve the levels of serum and colonic inflammatory markers, and restore the gut microbiota and metabolites	Fu et al. (2022)
Inulin	DSS-induced colitis in BALB/c mice	Reduces weight loss and the DAI	Qiao et al. (2022)
Inulin	Patients with UC	Increases the contents of colonic butyrate and the abundance of Bifidobacteriaceae and Lachnospiraceae	Valcheva et al. (2019)
Partially hydrolyzed guar gum	2,4,6-TNBS induced colitis in C57BL/6 mice	Regulates the gut microbiota and decreases TNF-α production and neutrophil infiltration	Takagi et al. (2016)
Nanoformulations of quercetin (QUE-B-GC micelles)	DSS-induced colitis in C57BL/6J mice	Suppress TNF-α, IL-6, and iNOS	Shen et al. (2021)
Nanoformulations of quercetin (QSFN)	DSS-induced colitis in C57BL/6J mice	Reduce the expression of pro-inflammatory cytokines (Tnf-α, Il-1β, Il-6, Mcp-1, Icam-1, Nlrp3, and iNOS)	Diez-Echave et al. (2021)
Nanoformulations of silybin	Acetic acid-induced colitis in rats	Reduce TNF-α, IL-6, and MPO activity	Varshosaz et al. (2015)
Nanoformulations of curcumin (PCur)	DSS-induced colitis in C57BL/6J mice	Ameliorate the inflammatory progression	Qiao et al. (2017)
Nanoformulations of curcumin	DSS-induced colitis in ICR mice	Reduce inflammation-related symptoms	Oshi et al. (2020)
Nanoformulations of curcumin (P-CUR/CAT-NPs)	DSS-induced colitis in FVB male mice	Inhibit the secretion of major pro-inflammatory cytokines	Huang et al. (2021a)
Nanoformulations of embelin (LNE)	Acetic acid-induced colitis in Wistar rats	Decrease MPO, LDH, and LPO levels	Badamaranahali et al. (2015)
Nanoformulations of embelin	DNBS-induced colitis in Wistar rats	Promote antioxidant and anti-inflammatory effects	Sharma et al. (2018)
Nanoparticles derived from edible ginger (GDNPs 2)	DSS-induced colitis in FVB/NJ mice	Decrease pro-inflammatory cytokines and increase anti-inflammatory cytokines	Zhang et al. (2016)
Grape-derived exosome-like nanoparticles (GELNs)	DSS-induced colitis in mice	Regulate the renewal process of intestinal tissue	Ju et al. (2013)
4-Aminothiophenol grafted onto carboxymethyl inulin as a delivery system for budesonide	DSS-induced colitis in BALB/c mice	Lowers the colon weight-to-length ratio and reduces weight loss and the DAI values	Sun et al. (2018)
		Presents the intact mucosal structure and reduces inflammatory cell infiltration	
Grafted polyacrylamide-grafted-xanthan gum copolymer as nanocarriers for curcumin	Acetate-induced colitis in Wistar rats	Decreases myeloperoxidase and nitrite contents, and alleviates body weight loss and colonic inflammation	Mutalik et al. (2016)

### 3.1 The well-known NPs with therapeutic effects in experimental UC models

Flavonoids have health-promoting properties and exhibit a wide range of biological activities (Xue J. et al., 2023). Several studies have shown that flavonoids have antioxidant and anti-inflammatory activities and are effective in treating UC. Park et al. indicated that kaempferol may reduce UC symptoms in mice with DSS-induced UC by modulating inflammatory biomarkers and protecting the colonic mucosa through arachidonic acid metabolism (Park et al., 2012). Quercetin has strong antioxidant activity and may improve intestinal inflammation by inhibiting cell apoptosis. Suzuki et al. demonstrated that quercetin enhances intestinal barrier function by increasing the expression of zona occludens--2, occludin, and claudin-4, thereby playing a crucial role in treating UC (Suzuki and Hara, 2009). Dou et al. demonstrated that isorhamnetin ameliorates chemically induced UC with its effects as a pregnane X receptor ligand. Isorhamnetin also alleviates weight loss and histological damage, promotes myeloperoxidase activity, and inhibits TNF-α and IL-6 levels (Dou et al., 2014). Epigallocatechin gallate (EGCG) has anti-inflammatory effects and promotes colon barrier integrity in DSS-induced UC mice. Utilizing fecal microbiota transplantation and the sterile fecal filtrate, Wu et al. demonstrated that EGCG ameliorated UC in a gut microbiota-dependent manner, specifically through SCFA-producing bacteria such as *Akkermansia* (Wu et al., 2021). The intake of isoflavones may alleviate UC-related clinical symptoms, particularly in reducing abdominal pain, suggesting that isoflavones could be a beneficial addition to the diets of UC patients (Skolmowska et al., 2019).

Non-flavonoid polyphenols, which have strong anti-inflammatory and antioxidant properties, are among the important secondary metabolites in plants and the most relevant natural antioxidants for humans. The polyphenol extracts from mature Pu-erh tea can alleviate colitis in mice, as shown by reduced pro-inflammatory cytokines and macrophage infiltration, and significant increases in the content of SCFAs and the expression of colonic peroxisome proliferator-activated receptor-γ (Huang et al., 2021a). Recent reports indicate that curcumin is a natural polyphenol exhibiting strong anti-inflammatory effects. Nuclear factor-κb (NF-κB) affects the mucosal inflammatory process of UC. Several studies have shown that curcumin can alleviate UC by inhibiting the expression of NF-κB (Wang et al., 2018). By reducing oxidative stress and inflammation, supplementation with resveratrol for 6 weeks improved disease clinical colitis activity and the quality of life in UC patients (Samsami-Kor et al., 2015).

Quinones are important secondary metabolites in plants. Juglone, extracted from *Juglans mandshurica*, exhibits anti-inflammatory activity, and can reduce the disease activity index and improve the pathological characteristics of UC mice. Therefore, it may protect mice against UC by regulating intestinal flora and Th17/Treg homeostasis (Hua et al., 2021). Tanshinone IIa can decrease DAI and histopathological scores, and the concentration of serum pro-inflammatory factors in UC. Tanshinone IIa affects UC in mice, specifically by regulating taurine and hypotaurine metabolism (Zhu et al., 2022). Terpenoids are a class of naturally occurring hydrocarbon compounds found widely in plants. Geraniol treatment of UC mice significantly reduced the DAI score, increased colon length, and reduced the pro-inflammatory cytokine content and myeloperoxidase activity. In addition, geraniol treatment also restored the decreased antioxidant parameters (Medicherla et al., 2015). Marius et al. isolated cyclocane-type triterpenoids from combretum fragrans. The effect of Combretin on UC induced by dextran sulfate sodium was studied. The results showed that Combretin effectively reduces DSS-induced colitis by inhibiting cyclooxygenase, 5-lipoxygenase, and protein denaturation, thus revealing the anti-inflammatory, antioxidative, and therapeutic properties of Combretin (Marius et al., 2020).

Natural alkaloids, specifically those derived from medicinal plants, exhibit a variety of pharmacological activities, including anti-inflammatory properties and immune regulation. Alkaloids from different sources have a significant inhibitory effect on UC. Chen et al. found that oxymatrine (OMT) extracted from the root of matrine has significant alleviating effects on UC. OMT improves UC through anti-inflammatory, pro-apoptotic, and PI3K/AKT pathways, suggesting that OMT is a promising drug for treating UC (Chen et al., 2017). The effects of berberrubine on UC were determined by the DSS-induced mouse model due to its anti-inflammatory and antibacterial effects. The results showed that berberrubine was found to recognize bitter taste receptors on intestinal tuft cells and promote the differentiation of intestinal stem cells to activate immune pathways (Xiong et al., 2021).

As functional carbohydrates, natural polysaccharides have a significant effect in UC therapy. The pectic polysaccharides AL-I from *Aconitum carmichaelii* leaves can alleviate the symptoms of DSS-induced ulcerative colitis in mice and improve the levels of serum and colonic inflammatory markers. The changes in the gut microbiota and the metabolites were restored (Fu et al., 2022). Inulin is an indigestible fructan mainly derived from Jerusalem artichoke and chicory. Inulin promotes the health of DSS-treated mice by reducing weight loss and the DAI (Qiao et al., 2022). Valcheva et al. suggested an inulin intake of 15 g/d for 9 weeks induced health benefits in UC patients (n = 13). Inulin increased the contents of colonic butyrate and the abundance of Bifidobacteriaceae and Lachnospiraceae in patients’ stool samples (Valcheva et al., 2019). Guar gum is a polysaccharide derived from the seeds of guar beans. Takagi et al. investigated the anti-inflammatory activities of partially hydrolyzed guar gum against 2,4,6-TNBS-induced colitis. The guar gum prevents colitis by regulating the gut microbiota (*Clostridium cluster* XIVa, *C. cluster* IV, and *Bacteroides fragilis*) and decreasing TNF-α production and neutrophil infiltration (Takagi et al., 2016).

### 3.2 Nanoformulations of NPs for UC treatment

Ineffective bioavailability is the primary limitation to NPs for treating UC. The advancement in nanoformulation procedures reduces the limitations to bioactive molecules, such as bioavailability, specificity, and solubility. Nanoformulations of NPs offer new prospects for treating UC.

Quercetin has been reported as a successful application of nanotechnology and has shown significant therapeutic potential in UC models. Shen et al. synthesized a prodrug micelle of active quercetin covalently linked to biocompatible ethylene glycol chitosan through aryl borate as a responsive linker. The micelles accumulated at the sites of intestinal inflammation and inhibited the expression of typical inflammatory factors. This work aims to improve the therapeutic efficacy of IBD by facilitating the inflammation-targeted delivery and intestinal drug accumulation of active single-agent quercetin (Shen et al., 2021). QSFN was prepared using quercetin-loaded silk fibroin nanoparticles, and its treatment showed intestinal anti-inflammatory properties in a mouse DSS model of colitis compared with the control group. QSFN significantly reduced UC disease activity index values. Histological examination of colon specimens and analysis of the expression of different pro-inflammatory cytokines in the colon confirmed beneficial effects. These data suggest that QSFN may be an attractive alternative therapy as a drug delivery system for treating IBD, providing support for using its quercetin in nanomedicine (Diez-Echave et al., 2021).

Silybin (SIL) is the primary and effective biological component of silymarin. Nanoparticles containing aqueous silibinin were prepared using Eudragit RL PO by solvent evaporative emulsification. Nanoparticles improved symptomatic and histopathological scores in UC. The nanoparticles significantly reduced the activities of TNF-α, IL-6, and MPO in rats with acetic acid-induced colitis compared to the control group (Varshosaz et al., 2015). Khurana et al. reported that combining silymarin and SeNP could attenuate experimentally induced colitis in rats caused by trinitrobenzene sulfonic acid (TNBS). The combination therapy showed better efficacy than SeNPs alone (Khurana et al., 2019).

Curcumin in the nanoformulation can increase the therapeutic effect. Qiao et al. synthesized an amphiphilic curcumin polymer (PCur) consisting of the hydrophilic polyethylene glycol and a hydrophobic curcumin linked by a disulfide bond. In the DSS-induced IBD mouse model, oral administration of PCur ameliorated inflammation progression in the colon and protected mice from IBD. The PCur conjugate may be used as a colon-specific candidate (Qiao et al., 2017). Oshi et al. reported the core–shell nanoparticles consisting of curcumin nanocapsules and chitosan/alginate multilayer films for UC. Compared to healthy tissues, nanoparticles preferentially accumulated in inflamed tissues, and in a mouse colitis model, nanoparticles were more effective at reducing inflammation-related symptoms (Oshi et al., 2020). Huang et al. used pluronic F127 to co-encapsulate catalase curcumin with functionalized polylactic acid-co-glycolic acid-based nanoparticles. The nanoparticles are more effective than pure natural active compounds at inhibiting the secretion of major pro-inflammatory cytokines (Huang et al., 2021b).

Embelin is the major bioactive component with antioxidant and anti-inflammatory activities derived from *Embelia ribes* Burm. f. Embelin LNs were prepared using a liquid lipid carrier of soybean oil/virgin coconut oil and a stabilizer of soybean/lecithin. The study utilized a rat model to induce UC through acetic acid. The results showed that embelin LN treatment reduced clinical activity and macroscopic scores significantly. Embelin LN treatment can also reduce MPO, LDH, and LPO levels (Badamaranahalli et al., 2015). Embelin-loaded guar gum particles were prepared using an emulsification technique. Due to its antioxidant and anti-inflammatory effects, embelin pretreatment can prevent colitis and improve its symptoms (Sharma et al., 2018).

Plant-derived nanoparticles can act as exosomes and participate in cell-to-cell communication. Zhang et al. reported edible ginger (GDNPs 2) nanoparticles. Nontoxic GDNPs 2 were predominantly absorbed by the intestinal epithelial cells (Iecs) and macrophages. The administration of GDNPs 2 orally increased IEC survival and proliferation, decreased pro-inflammatory cytokines, and increased anti-inflammatory cytokines. These results suggest that GDNPs 2 may reduce harmful factors and facilitate healing (Zhang et al., 2017). Ju et al. identified the exosome-like nanoparticles from grapes. Grape-derived exosome-like nanoparticles (GELN) target intestinal stem cells and form the basis for intestinal tissue remodeling and protective effects against colitis by DSS. GELN can regulate the renewal process of intestinal tissue and participate in the remodeling of intestinal tissue triggered by pathology (Ju et al., 2013).

Polysaccharides can be used as nanocarriers to carry effective drugs for the targeted therapy of UC. Budesonide is a synthetic steroid hormone that can be used in the inflammatory state of UC. Sun et al. designed a nanocarrier with 4-aminothiophenol grafted onto carboxymethyl inulin as a delivery system for budesonide. The nanoparticles had the average particle size ∼210.18 nm. The nanoparticles can accumulate at the inflammation sites of colon. Excellent therapeutic effects were observed compared with the single budesonide suspension in the colitis mouse model (Sun et al., 2018). Mutalik et al. obtained a grafted polyacrylamide-grafted xanthan gum copolymer as nanocarriers for curcumin. The nanoparticles were spherical with an average particle size of 425 nm. The nanoparticles decreased myeloperoxidase and nitrite contents, and alleviated body weight loss and colonic inflammation in rats with acetate-induced colitis. In addition, curcumin in the nanoparticle form showed better systemic absorption with increased C_max_ (3-fold) and AUC (2.5-fold) than free curcumin (Mutalik et al., 2016).

## 4 Nanoparticles–gut microbiota interference

**FIGURE 1:**
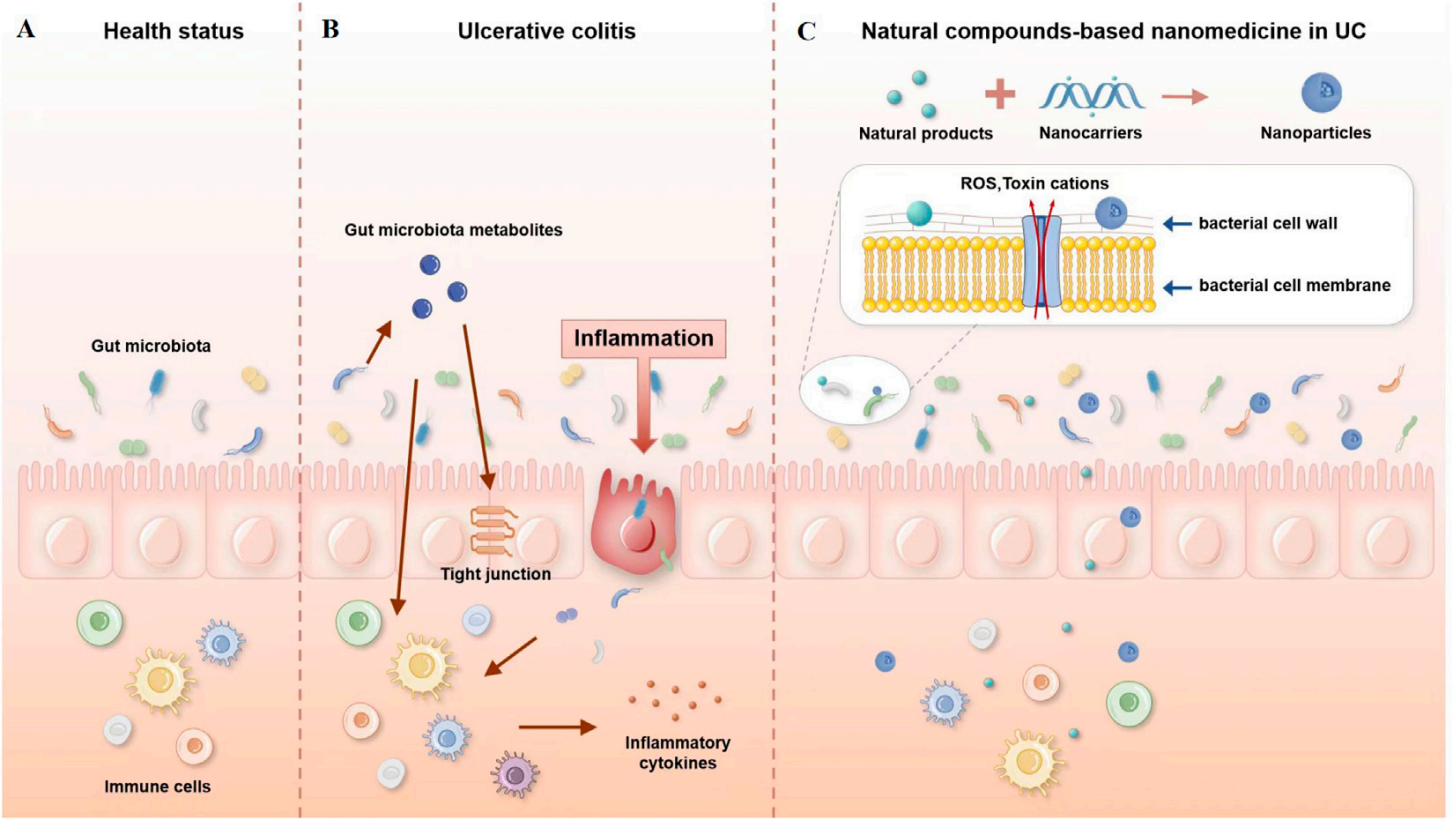
Possible mechanisms of interaction between nanoparticles and the gut microbiota. **(A)** Healthy gut, **(B)** gut with ulcerative colitis, and **(C)** natural compound-based nanomedicine in ulcerative colitis therapy.

Once nanoparticles enter the intestinal lumen, there is a high potential for contact with various microorganisms. The interaction between nanoparticles and the gut microbiota is bidirectional. Intestinal microorganisms can have their structure and metabolic function regulated by nanoparticles. On the other hand, nanoparticles will be affected by intestinal flora, which may increase or decrease their effectiveness. The possible mechanisms are shown in Figure 1.

### 4.1 Influence of nanoparticles on the gut microbiota

The nanoparticles can be used to restore gut microbiota balance in UC therapy. Orally administered amyloid–polyphenol hydrogel can remain in the colon for an extended time. The hydrogel had a significant positive effect on colitis mouse models, improving intestinal barrier function and regulating intestinal flora imbalance by reducing the abundance of operational taxa that is normally enriched for colitis, specifically facultative anaerobes such as *Aestuariispira* and *Escherichia*. Additionally, the short-chain fatty acid metabolites were enriched (Hu et al., 2020). In a mouse model of acute colitis, Lee et al. constructed a hyaluronic acid–bilirubin nanomedicine (HABN) that targets the inflamed colon and restores the epithelial barrier. HABN regulates intestinal flora by increasing the overall richness and diversity, and significantly increasing the number of microorganisms that play an essential role in intestinal homeostasis, such as *Akkermansia muciniphila* and *Clostridium XIVa* (Lee et al., 2020). Alfaro-Viquez et al. synthesized a hybrid nanoparticle containing cranberry proanthocyanidin–chitosan (PAC-CHTNp) and studied its effect on the invasion of intestinal epithelial cells ExPEC. The results showed that PAC-CHTNp significantly inhibited the invasion of intestinal epithelial cells and can improve the stability of PAC and promote the molecular adhesion of PAC to ExPEC (Alfaro-Viquez et al., 2018). Glycogen modified with urocanic acid and α-lipoic acid could form nanoparticles by self. The nanocarrier could encapsulate ginsenoside Rh2 to form Rh2 nanoparticles (Rh2 NPs). Rh2 NPs could improve the inflammation conditions and histological scores in UC mice. Rh2 NPs can also restore the diversity of intestinal flora and play beneficial roles (Xu et al., 2022).

Studies have shown that plant-derived NPs are effective weapons against specific strains. Zhang et al. found that oral administration of ginger lipid nanoparticles (GDLPs) can target *L. rhamnosus* GG. In mice with colitis, mdo-miR7267-3p, a microRNA of GDLPs, targets *L. rhamnosus* GG. monooxygenase to increase the production of indole 3-carboxyaldehyde and improve the intestinal barrier function (Zhang et al., 2016). Tong et al. found that mEVs contain many immunoactive proteins and regulate intestinal immunity and microbiota in mice. Oral administration of meV prevented colon shortening and reduced intestinal epithelial destruction in a mouse model of UC. The intestinal flora was also partially recovered after meV intervention, suggesting that meV may regulate intestinal immunity by affecting the intestinal flora (Tong et al., 2021).

Nanoparticles can inhibit bacteria through different ways, such as destabilizing bacterial cells, producing reactive oxygen species, inhibiting protein synthesis, and releasing toxic cations (Ladaycia et al., 2021). There are many factors that influence the antibacterial activities. The smaller nanoparticles might have better chances to interact with the bacterial cell membrane for bacterial toxicity (Applerot et al., 2009). The shape and size of nanoparticles may also affect the surface area in contact with bacteria, which may affect the bactericidal efficiency. The antibacterial activity of quercetin in nanocarriers was higher than that of quercetin. The nanoformulations were found to be more sensitive against Gram-negative (G -ve) bacteria (*E. coli* and *P. denitrificans*). The antibacterial activity depended on time and concentration (Das et al., 2020). Another report revealed that quercetin-loaded alginate/chitosan nanoparticles had more antibacterial activities than pure quercetin (Nalini et al., 2022).

The antibacterial activity of nanoparticles also depends on the bacteria type (Gram-positive or Gram-negative). Gram-positive bacteria have a thick peptidoglycan layer with polymer teichoic acids and a cytoplasmic membrane underneath, whereas Gram-negative bacteria have two lipid membranes with a thin peptidoglycan layer in between. Both bacterial wall membranes are negatively charged, regardless of the various functional groups on their surface (Feng et al., 2015). The Gram-negative bacterial cell wall is known to be more resistant to the antibacterial effect because it is harder to cross (Graef et al., 2018). The mechanisms of nanoparticles modulating the gut microbiota are still in the initial stage. Antibacterial activity reports suggest that nanoparticles may destroy, shift, repair, or treat the human microbiota by targeting and acting with specific gut microbiota.

### 4.2 Gut microbiota affects the nanoparticles

Polymer materials, such as pectin, chitosan, and dextran, are used as nanocarriers. They are stable in the upper GI tract. However, they can be degraded by specific enzymes (β-glucosidase, cellulase, and azoreductase) produced by the gut microbiota. Based on this property, we can design the microbial-triggered nano-delivery system (Tang et al., 2020). Chen et al. developed a nano-drug delivery system based on the chitosan-modified, Dex-loaded, esterase-responsive lipid with 3,3′-dithiodipropionic acid, quercetin, and glyceryl caprylate–caprate. In esterase-containing artificial intestinal fluid, the nanoparticles could rapidly release the drug. The nanoparticles could reduce pro-inflammatory cytokine expression, colonic atrophy, and histomorphological changes in the DSS-induced colitis mouse model while increasing E-cadherin expression (Chen et al., 2020). The gut microbiota could also affect nanomaterial absorption. The lipopolysaccharides (LPS) produced by Gram-negative bacteria in the colon provide extra adherence for nanoparticles, resulting in the enhanced absorption of nanomedicine (Cattani et al., 2010; Xue JC. et al, 2023).

The gut microbiota may have harmful or beneficial effects on the metabolism of nanomedicines. The gut microbiota can metabolize a variety of natural active compounds, and this metabolism may lead to lower absorption and bioavailability of natural active products. Bacterial species, such as *Bifidobacterium* sp., *Lactobacillus* sp., and *Eubacterium* sp., could catalyze phenolic metabolism (Duda-Chodak, 2012; Shabbir et al., 2021). *E. coli*, *E. fergusonii* (ATCC 35469), *Bifidobacterium*, and *Lactobacillus* play a vital role in the degradation of curcumin (Zam, 2018). *Bacteroides fragilis*, *Eubacterium ramulus*, and *Clostridium perfringens* are the bacterial strains that transform quercetin into metabolites (Zhang et al., 2014). The bioactive components can be encapsulated in nanocarriers to prevent metabolism by intestinal flora and maintain stability. The gut microbiota is capable of biotransforming the nanodrugs released in the colon to promote the treatment of colitis. The gut microbiota can degrade anthocyanins to phloroglucinol derivatives and benzoic acids (Luo et al., 2019; Rosales et al., 2022). The metabolites stimulate or inhibit the growth of other specific bacteria, thereby further regulating the gut microbiota. Anthocyanin metabolites can promote the production of SCFAs, reduce intestinal pH, and inhibit pathogen growth (Tian, et al., 2019).

Most polysaccharide-based drug nanocarrier systems (DNSs) release drugs in the colon successfully. The gut microbiota plays an important role in the breakdown of polysaccharide-based DNSs. Polysaccharides will cooperate with released drugs to treat UC. The gut microbiota can encode abundant carbohydrate-active enzymes that convert polysaccharides into useable monosaccharides for beneficial bacteria (Zheng et al., 2020). The degraded polysaccharides can further be metabolized by the gut microbiota into beneficial products, such as SCFAs (Cui et al., 2021). For example, the *Ficus carica* polysaccharide can increase the abundance of S24-7, *Bacteroides,* and *Coprococcus*, as well as the levels of acetate and butyric acid, which has a protective effect on UC (Zou et al., 2020).

Natural carbon nanoparticles isolated from beer can also be adsorbed by bacteria to form complexes. Nanoparticle–bacterial complexes affect phagocyte uptake, pathogenic signaling pathways, and nanoparticle-induced cytotoxicity. Nanoparticles can be harnessed to (reasonably) shape the microbiome and inhibit pathogenic bacteria (Siemer et al., 2018). Although some progress has been made in understanding the effects of nanomaterials on the gut microbiota, the precise mechanisms by which nanomaterials affect the gut microbiota remain unclear. Further research is required to assess the effects of nanomaterials on the microbiota.

## 5 Conclusion and future perspectives

Natural products can be used as potential therapeutic methods for treating human UC due to their clear efficacy and low adverse reactions. Unfortunately, low oral bioavailability has limited using natural compounds in clinical trials. Nanoformulations are an effective way to improve the bioavailability of natural compounds. Nanotechnology can deliver effective therapeutic agents to specific body areas while controlling the release of natural bioactive metabolites. This review summarizes the great potential of natural products and their nanoparticles for preventing, managing, and treating UC.

Dysbiosis of intestinal flora is associated with the pathogenesis of many diseases. Targeting the gut microbiota is also applicable to the intervention of a variety of diseases. Oral nanomedicine provides a feasible strategy to modulate the gut microbiota and metabolites. The possible mechanisms could be summarized. Natural compounds can be encapsulated by nanocarriers to prolong the action time and improve the activity, or they can be used as nanocarriers to deliver drugs to play the dual role of regulating the flora and improving the activity of drugs. The interaction between nanomaterials and intestinal flora can improve the efficacy of nanomedicine. Recent advances in nanomedicine delivery systems constructed in recent years will facilitate the clinical application of nanomedicines in preventing or treating UC. Although nanomedicines have advanced significantly in the last decade, there are still scientific and technical challenges. As a result, there is growing interest in natural product-based nanomedicines for UC treatment.

## References

[B1] Al-Bayati L. Nayeri Fasaei B. Merat S. Bahonar A. (2018). Longitudinal analyses of gut-associated bacterial microbiota in ulcerative colitis patients. Arch. Iran. Med. 21 (12), 578–584. 30634855

[B2] Alfaro-Viquez E. Esquivel-Alvarado D. Madrigal-Carballo S. Krueger C. G. Reed J. D. (2018). Cranberry proanthocyanidin-chitosan hybrid nanoparticles as a potential inhibitor of extra-intestinal pathogenic *Escherichia coli* invasion of gut epithelial cells. Int. J. Biol. Macromol. 111, 415–420. 10.1016/j.ijbiomac.2018.01.033 29325748

[B3] Applerot G. Lipovsky A. Dror R. Perkas N. Nitzan Y. Lubart R. (2009). Enhanced antibacterial activity of nanocrystalline Zn O due to increased ROSmediated cell injury. Adv. Func. Mater 19 (6), 842–852. 10.1002/adfm.200801081

[B4] Badamaranahalli S. S. Kopparam M. Bhagawati S. T. Durg S. (2015). Embelin lipid nanospheres for enhanced treatment of ulcerative colitis–Preparation, characterization and *in vivo* evaluation. Eur. J. Pham Sci. 76, 73–82. 10.1016/j.ejps.2015.05.003 25957524

[B5] Barberio B. Facchin S. Patuzzi I. Ford A. C. Massimi D. Valle G. (2022). A specific microbiota signature is associated to various degrees of ulcerative colitis as assessed by a machine learning approach. Gut Microbes 14 (1), 2028366. 10.1080/19490976.2022.2028366 35129058 PMC8820804

[B6] Bian X. Wu W. Yang L. Lv L. Wang Q. Li Y. (2019). Administration of *Akkermansia muciniphila* ameliorates dextran sulfate sodium-induced ulcerative colitis in mice[J]. Front. Microbiol. 10, 2259. 10.3389/fmicb.2019.02259 31632373 PMC6779789

[B7] Bischoff S. C. Bager P. Escher J. Forbes A. Hébuterne X. Hvas C. L. (2023). ESPEN guideline on Clinical Nutrition in inflammatory bowel disease. Clin. Nutr. 42 (3), 352–379. 10.1016/j.clnu.2022.12.004 36739756

[B8] Bjerrum J. T. Wang Y. Hao F. Coskun M. Ludwig C. Günther U. (2015). Metabonomics of human fecal extracts characterize ulcerative colitis, Crohn's disease and healthy individuals. Metabolomics 11, 122–133. 10.1007/s11306-014-0677-3 25598765 PMC4289537

[B9] Browne H. P. Forster S. C. Anonye B. O. Kumar N. Neville B. A. Stares M. D. (2016). Culturing of “unculturable”human microbiota reveals novel taxa and extensive sporulation. Nature 533, 543–546. 10.1038/nature17645 27144353 PMC4890681

[B10] Burrello C. Garavaglia F. Cribiù F. M. Ercoli G. Lopez G. Troisi J. (2018). Therapeutic faecal microbiota transplantation controls intestinal inflammation through IL10 secretion by immune cells. Nat. Commun. 9 (1), 5184. 10.1038/s41467-018-07359-8 30518790 PMC6281577

[B11] Cao F. Gui S. Y. Gao X. Zhang W. Fu Z. Y. Tao L. M. (2022). Research progress of natural product-based nanomaterials for the treatment of inflammation-related diseases. Mater Des. 218, 110686. 10.1016/j.matdes.2022.110686

[B12] Caruso R. Lo B. C. Núñez G. (2020). Host-microbiota interactions in inflammatory bowel disease. Nat. Rev. Immunol. 20 (7), 411–426. 10.1038/s41577-019-0268-7 32005980

[B13] Cattani V. B. Fiel L. A. Jäger A. Jäger E. Colomé L. M. Uchoa F. (2010). Lipid-core nanocapsules restrained the indomethacin ethyl ester hydrolysis in the gastrointestinal lumen and wall acting as mucoadhesive reservoirs. Eur. J. Pharm. Sci. 39, 116–124. 10.1016/j.ejps.2009.11.004 19932749

[B14] Chen Q. Duan X. Fan H. Xu M. Tang Q. Zhang L. (2017). Oxymatrine protects against DSS-induced colitis via inhibiting the PI3K/AKT signaling pathway. Int. Immunopharmacol. 53, 149–157. 10.1016/j.intimp.2017.10.025 29107215

[B15] Chen S. Q. Song Y. Q. Wang C. Tao S. Yu F. Y. Lou H. Y. (2020). Chitosan-modified lipid nanodrug delivery system for the targeted and responsive treatment of ulcerative colitis. Carbohydr. Polym. 230, 115613. 10.1016/j.carbpol.2019.115613 31887935

[B17] Chibbar R. Dieleman L. A. (2015). Probiotics in the management of ulcerative colitis. J. Clin. Gastroenterol. 49 (1), 50–55. 10.1097/mcg.0000000000000368 26447965

[B112] Cui L. Guan X. Ding W. Luo Y. Wang W. Bu W. (2021). Scutellaria baicalensis Georgi polysaccharide ameliorates DSS-induced ulcerative colitis by improving intestinal barrier function and modulating gut microbiota. Int. J. Biol. Macromol. 166, 1035–1045. 10.1016/j.ijbiomac.2020.10.259 33157130

[B18] Dahal R. H. Kim S. Kim Y. K. Kim E. S. Kim J. (2023). Insight into gut dysbiosis of patients with inflammatory bowel disease and ischemic colitis. Front. Microbiol. 14, 1174832. 10.3389/fmicb.2023.1174832 37250025 PMC10211348

[B19] Das S. S. Verma P. R. P. Singh S. K. (2020). Screening and preparation of quercetin doped nanoemulsion: Characterizations, antioxidant and anti-bacterial activities. LWT 124, 109141. 10.1016/j.lwt.2020.109141

[B20] Diez-Echave P. Ruiz-Malagón A. J. Molina-Tijeras J. A. Hidalgo-García L. Vezza T. Cenis-Cifuentes L. (2021). Silk fibroin nanoparticles enhance quercetin immunomodulatory properties in DSS-induced mouse colitis. Int. J. Pharm. 606, 120935. 10.1016/j.ijpharm.2021.120935 34310954

[B21] Dou W. Zhang J. Li H. Kortagere S. Sun K. Ding L. (2014). Plant flavonol isorhamnetin attenuates chemically induced inflammatory bowel disease via a PXR-dependent pathway. J. Nutr. Biochem. 25 (9), 923–933. 10.1016/j.jnutbio.2014.04.006 24913217 PMC4125479

[B22] Du L. Ha C. (2020). Epidemiology and pathogenesis of ulcerative colitis. Gastroenterol. Clin. North Am. 49 (4), 643–654. 10.1016/j.gtc.2020.07.005 33121686

[B23] Duda-Chodak A. (2012). The inhibitory effect of polyphenols on human gut microbiota. J. Physiol. Pharm. 63, 497–503. 23211303

[B24] Feng Z. V. Gunsolus I. L. Qiu T. A. Hurley K. R. Nyberg L. H. Frew H. (2015). Impacts of gold nanoparticle charge and ligand type on surface binding and toxicity to Gram-negative and Gram-positive bacteria. Chem. Sci. 6 (9), 5186–5196. 10.1039/c5sc00792e 29449924 PMC5669217

[B25] Fite A. Macfarlane S. Furrie E. Bahrami B. Cummings J. H. Steinke D. T. (2013). Longitudinal analyses of gut mucosal microbiotas in ulcerative colitis in relation to patient age and disease severity and duration. J. Clin. Microbiol. 51 (3), 849–856. 10.1128/JCM.02574-12 23269735 PMC3592070

[B26] Fu Y. P. Li C. Y. Peng X. Wangensteen H. Inngjerdingen K. T. Zou Y. F. (2022). Pectic polysaccharides from Aconitum carmichaelii leaves protects against DSS-induced ulcerative colitis in mice through modulations of metabolism and microbiota composition. Biomed. Pharmacother. 55, 113767. 10.1016/j.biopha.2022.113767 36271551

[B27] Gajendran M. Loganathan P. Jimenez G. Catinella A. P. Ng N. Umapathy C. (2019). A comprehensive review and update on ulcerative colitis. Dis. Mon. 65 (12), 100851. 10.1016/j.disamonth.2019.02.004 30837080

[B28] Galipeau H. J. Caminero A. Turpin W. Bermudez-Brito M. Santiago A. Libertucci J. (2021). Novel fecal biomarkers that precede clinical diagnosis of ulcerative colitis. Gastroenterology 160 (5), 1532–1545. 10.1053/j.gastro.2020.12.004 33310084

[B29] Ghosh S. Whitley C. S. Haribabu B. Jala V. R. (2021). Regulation of intestinal barrier function by microbial metabolites. Cell Mol. Gastroenter 11 (5), 1463–1482. 10.1016/j.jcmgh.2021.02.007 PMC8025057 33610769

[B30] Graef F. Richter R. Fetz V. Murgia X. De Rossi C. Schneider-Daum N. (2018). *In vitro* model of the gram-negative bacterial cell envelope for investigation of anti-infective permeation kinetics. ACS Infect. Dis. 4 (8), 1188–1196. 10.1021/acsinfecdis.7b00165 29750862

[B31] Guo X. Y. Liu X. J. Hao J. Y. (2020). Gut microbiota in ulcerative colitis: Insights on pathogenesis and treatment. J. Dig. Dis. 21 (3), 147–159. 10.1111/1751-2980.12849 32040250

[B32] Hosseinkhani F. Heinken A. Thiele I. Lindenburg P. W. Harms A. C. Hankemeier T. (2021). The contribution of gut bacterial metabolites in the human immune signaling pathway of non-communicable diseases. Gut Micro 13, 1–22. 10.1080/19490976.2021.1882927 PMC7899087 33590776

[B33] Hu B. Yu S. Shi C. Gu J. Shao Y. Chen Q. (2020). Amyloid–polyphenol hybrid nanofilaments mitigate colitis and regulate gut microbial dysbiosis. ACS Nano 14 (3), 2760–2776. 10.1021/acsnano.9b09125 31961657

[B34] Hua Y. Liu R. Lu M. Guan X. Zhuang S. Tian Y. (2021). Juglone regulates gut microbiota and Th17/Treg balance in DSS-induced ulcerative colitis. Int. Immunopharmacol. 97, 107683. 10.1016/j.intimp.2021.107683 33915494

[B35] Huang Y. Canup B. S. B. Gou S. Chen N. Dai F. Xiao B. (2021a). Oral nanotherapeutics with enhanced mucus penetration and ROS-responsive drug release capacities for delivery of curcumin to colitis tissues. J. Mater Chem. B 9 (6), 1604–1615. 10.1039/d0tb02092c 33471012

[B36] Huang Y. Yang Q. Mi X. Qiu L. Tao X. Zhang Z. (2021b). Ripened Pu-erh tea extract promotes gut microbiota resilience against dextran sulfate sodium induced colitis. J. Agric. Food Chem. 69 (7), 2190–2203. 10.1021/acs.jafc.0c07537 33570405

[B37] Ishikawa D. Sasaki T. Takahashi M. Kuwahara-Arai K. Haga K. Ito S. (2018). The microbial composition of Bacteroidetes species in ulcerative colitis is effectively improved by combination therapy with fecal microbiota transplantation and antibiotics. Inflamm. Bowel Dis. 24 (12), 2590–2598. 10.1093/ibd/izy266 30124831

[B38] Jalanka J. Cheng J. Hiippala K. Ritari J. Salojärvi J. Ruuska T. (2020). Colonic mucosal microbiota and association of bacterial taxa with the expression of host antimicrobial peptides in pediatric ulcerative colitis. Int. J. Mol. Sci. 21 (17), 6044. 10.3390/ijms21176044 32842596 PMC7504357

[B39] Jethwani P. Grover K. (2019). Gut microbiota in health and diseases—A review. Int. J. Curr. Microbiol. Appl. Sci. 8 (8), 1586–1599. 10.20546/ijcmas.2019.808.187

[B40] Ju S. Mu J. Dokland T. Zhuang X. Wang Q. Jiang H. (2013). Grape exosome-like nanoparticles induce intestinal stem cells and protect mice from DSS-induced colitis. Mol. Ther. 21 (7), 1345–1357. 10.1038/mt.2013.64 23752315 PMC3702113

[B41] Khurana A. Tekula S. Saifi M. A. Venkatesh P. Godugu C. (2019). Therapeutic applications of selenium nanoparticles. Biomed. Pharmacother. 111, 802–812. 10.1016/j.biopha.2018.12.146 30616079

[B42] Knoll R. L. Forslund K. Kultima J. R. Meyer C. U. Kullmer U. Sunagawa S. (2017). Gut microbiota differs between children with inflammatory bowel disease and healthy siblings in taxonomic and functional composition: A metagenomic analysis. Am. J. Physiol. Gastrointest. Liver Physiol. 312 (4), 327–339. 10.1152/ajpgi.00293.2016 28039159

[B43] Kudelka M. R. Stowell S. R. Cummings R. D. Neish A. S. (2020). Intestinal epithelial glycosylation in homeostasis and gut microbiota interactions in IBD. Nat. Rev. Gastroenterol. Hepatol. 17, 597–617. 10.1038/s41575-020-0331-7 32710014 PMC8211394

[B44] Ladaycia A. Passirani C. Lepeltier E. (2021). Microbiota and nanoparticles: Description and interactions. Eur. J. Pharm. Biopharm. 169, 220–240. 10.1016/j.ejpb.2021.10.015 34736984

[B45] Lajczak-McGinley N. K. Porru E. Fallon C. M. Smyth J. Curley C. McCarron P. A. (2020). The secondary bile acids, ursodeoxycholic acid and lithocholic acid, protect against intestinal inflammation by inhibition of epithelial apoptosis. Physiol. Rep. 8, e14456. 10.14814/phy2.14456 32562381 PMC7305237

[B114] Lang A. Salomon N. Wu J. C. Kopylov U. Lahat A. Har-Noy O. (2015). Curcumin in combination with mesalamine induces remission in patients with mild-to-moderate ulcerative colitis in a randomized controlled trial. Clin. Gastroenterol Hepatol. 13 (8), 1444–1449. 10.1016/j.cgh.2015.02.019 25724700

[B46] Lavelle A. Lennon G. O'Sullivan O. Docherty N. Balfe A. Maguire A. (2015). Spatial variation of the colonic microbiota in patients with ulcerative colitis and control volunteers. Gut 64 (10), 1553–1561. 10.1136/gutjnl-2014-307873 25596182 PMC4602252

[B47] Lee Y. Sugihara K. Gillilland M. G. Jon S. Kamada N. Moon J. J. (2020). Hyaluronic acid-bilirubin nanomedicine for targeted modulation of dysregulated intestinal barrier, microbiome and immune responses in colitis. Nat. Mater 19 (1), 118–126. 10.1038/s41563-019-0462-9 31427744 PMC6923573

[B48] Lenoir M. Martín R. Torres-Maravilla E. Chadi S. González-Dávila P. Sokol H. (2020). Butyrate mediates anti-inflammatory effects of*Faecalibacterium prausnitzii* in intestinal epithelial cells through *Dact3* . Gut Microbes 12 (1), 1–16. 10.1080/19490976.2020.1826748 PMC7567499 33054518

[B49] Li M. van Esch B. Henricks P. A. J. Garssen J. Folkerts G. (2021). IL-33 is involved in the anti-inflammatory effects of butyrate and propionate on TNFa-activated endothelial cells. Int. J. Mol. Sci. 22 (5), 2447. 10.3390/ijms22052447 33671042 PMC7957702

[B50] Luo Y. Fang J. L. Yuan K. Jin S. H. Guo Y. (2019). Ameliorative effect of purified anthocyanin from *Lycium ruthenicum* on atherosclerosis in rats through synergistic modulation of the gut microbiota and NF-κB/SREBP-2 pathways. J. Funct. Foods 59, 223–233. 10.1016/j.jff.2019.05.038

[B51] Machiels K. Joossens M. Sabino J. De Preter V. Arijs I. Eeckhaut V. (2014). A decrease of the butyrate-producing species *Roseburia hominis* and *Faecalibacterium prausnitzii* defines dysbiosis in patients with ulcerative colitis. Gut 63 (8), 1275–1283. 10.1136/gutjnl-2013-304833 24021287

[B52] Mar J. S. LaMere B. J. Lin D. L. Levan S. Nazareth M. Mahadevan U. (2016). Disease severity and immune activity relate to distinct interkingdom gut microbiome states in ethnically distinct ulcerative colitis patients. mBio 7 (4), e01072–16. 10.1128/mBio.01072-16 27531910 PMC4992973

[B53] Marius M. Amadou D. Donatien A. A. Gilbert A. William Y. N. Rauf K. (2020). *In vitro* antioxidant, anti-inflammatory, and *in vivo* anticolitis effects of combretin A and combretin B on dextran sodium sulfate-induced ulcerative colitis in mice. Gastroenterol. Res. Pract. 2020, 4253174. 10.1155/2020/4253174 33204254 PMC7666632

[B54] McIlroy J. Ianiro G. Mukhopadhya I. Hansen R. Hold G. L. (2018). Review article: The gut microbiome in inflammatory bowel disease-avenues for microbial management. Aliment. Pharmacol. Ther. 47 (1), 26–42. 10.1111/apt.14384 29034981

[B55] Medicherla K. Sahu B. D. Kuncha M. Kumar J. M. Sudhakar G. Sistla R. (2015). Oral administration of geraniol ameliorates acute experimental murine colitis by inhibiting pro-inflammatory cytokines and NF-κB signaling. Food Funct. 6 (9), 2984–2995. 10.1039/c5fo00405e 26190278

[B56] Mills R. H. Dulai P. S. Vázquez-Baeza Y. Sauceda C. Daniel N. Gerner R. R. (2022). Multi-omics analyses of the ulcerative colitis gut microbiome link *Bacteroides vulgatus* proteases with disease severity. Nat. Microbiol. 7, 262–276. 10.1038/s41564-021-01050-3 35087228 PMC8852248

[B57] Miquel S. Leclerc M. Martin R. Chain F. Lenoir M. Raguideau S. (2015). Identification of metabolic signatures linked to anti-inflammatory effects of *Faecalibacterium prausnitzii* . mBio 6 (2), e00300–e00315. 10.1128/mBio.00300-15 25900655 PMC4453580

[B58] Mukherjee A. Lordan C. Ross R. P. Cotter P. D. (2020). Gut microbes from the phylogenetically diverse genus Eubacterium and their various contributions to gut health. Gut Microbes 12 (1), 1802866. 10.1080/19490976.2020.1802866 32835590 PMC7524325

[B59] Mutalik S. Suthar N. A. Managuli R. S. Shetty P. K. Avadhani K. Kalthur G. (2016). Development and performance evaluation of novel nanoparticles of a grafted copolymer loaded with curcumin. Int. J. Biol. Macromol. 86, 709–720. 10.1016/j.ijbiomac.2015.11.092 26851203

[B60] Nalini T. Basha S. K. Sadiq A. M. Kumari V. S. (2022). *In vitro* cytocompatibility assessment and antibacterial effects of quercetin encapsulated alginate/chitosan nanoparticle. Int. J. Biol. Macromol. 219, 304–311. 10.1016/j.ijbiomac.2022.08.007 35934075

[B61] Ng S. C. Shi H. Y. Hamidi N. Underwood F. E. Tang W. Benchimol E. I. (2017). Worldwide incidence and prevalence of inflammatory bowel disease in the 21st century: A systematic review of population-based studies. Lancet 390 (10114), 2769–2778. 10.1016/S0140-6736(17)32448-0 29050646

[B62] Nomura K. Ishikawa D. Okahara K. Ito S. Haga K. Takahashi M. (2021). Bacteroidetes species are correlated with disease activity in ulcerative colitis. J. Clin. Med. 10 (8), 1749. 10.3390/jcm10081749 33920646 PMC8073534

[B63] Ohkusa T. Koido S. (2015). Intestinal microbiota and ulcerative colitis. J. Infect. Chemother. 21 (11), 761–768. 10.1016/j.jiac.2015.07.010 26346678

[B64] Oshi M. A. Lee J. Naeem M. Hasan N. Kim J. Kim H. J. (2020). Curcumin nanocrystal/pH-responsive polyelectrolyte multilayer core–shell nanoparticles for inflammation-targeted alleviation of ulcerative colitis. Biomacromolecules 21 (9), 3571–3581. 10.1021/acs.biomac.0c00589 32701266

[B65] Parada Venegas D. De la Fuente M. K. Landskron G. González M. J. Quera R. Dijkstra G. (2019). Short chain fatty acids (SCFAs)-mediated gut epithelial and immune regulation and its relevance for inflammatory bowel diseases. Front. Immunol. 10, 277. 10.3389/fimmu.2019.00277 30915065 PMC6421268

[B66] Park M. Y. Ji G. E. Sung M. K. (2012). Dietary kaempferol suppresses inflammation of dextran sulfate sodium-induced colitis in mice. Dig. Dis. Sci. 57, 355–363. 10.1007/s10620-011-1883-8 21901258

[B67] Passos M. D. C. F. Moraes-Filho J. P. (2017). Intestinal microbiota in digestive diseases. Arq. Gastroenterol. 54 (3), 255–262. 10.1590/S0004-2803.201700000-31 28723981

[B68] Qiao H. Fang D. Chen J. Sun Y. Kang C. Di L. (2017). Orally delivered polycurcumin responsive to bacterial reduction for targeted therapy of inflammatory bowel disease. Drug Deliv. 24 (1), 233–242. 10.1080/10717544.2016.1245367 28156160 PMC8240970

[B69] Qiao H. Zhao T. Yin J. Zhang Y. Ran H. Chen S. (2022). Structural characteristics of inulin and microcrystalline cellulose and their effect on ameliorating colitis and altering colonic microbiota in dextran sodium sulfate-induced colitic mice. ACS Omega 7 (13), 10921–10932. 10.1021/acsomega.1c06552 35415348 PMC8991927

[B111] Rajendran K. Karthikeyan A. Krishnan U. M. (2022). Emerging trends in nano-bioactive-mediated mitochondria-targeted therapeutic stratagems using polysaccharides, proteins and lipidic carriers. Int. J. Biol. Macromol. 208, 627–641. 10.1016/j.ijbiomac.2022.03.121 35341885

[B70] Rooks M. Garrett W. (2016). Gut microbiota, metabolites and host immunity. Nat. Rev. Immunol. 16, 341–352. 10.1038/nri.2016.42 27231050 PMC5541232

[B71] Rosales T. K. O. Hassimotto N. M. A. Lajolo F. M. Fabi J. P. (2022). Nanotechnology as a tool to mitigate the effects of intestinal microbiota on metabolization of anthocyanins. Antioxidants 11 (3), 506. 10.3390/antiox11030506 35326155 PMC8944820

[B72] Ryan F. J. Ahern A. M. Fitzgerald R. S. Laserna-Mendieta E. J. Power E. M. Clooney A. G. (2020). Colonic microbiota is associated with inflammation and host epigenomic alterations in inflammatory bowel disease. Nat. Commun. 11 (1), 1512. 10.1038/s41467-020-15342-5 32251296 PMC7089947

[B73] Samsami-Kor M. Daryani N. E. Asl P. R. Hekmatdoost A. (2015). Anti-inflammatory effects of resveratrol in patients with ulcerative colitis: A randomized, double-blind, placebo-controlled pilot study. Arch. Med. Res. 46 (4), 280–285. 10.1016/j.arcmed.2015.05.005 26002728

[B74] Santoru M. L. Piras C. Murgia A. Palmas V. Camboni T. Liggi S. (2017). Cross sectional evaluation of the gut-microbiome metabolome axis in an Italian cohort of IBD patients. Sci. Rep. 7, 9523. 10.1038/s41598-017-10034-5 28842640 PMC5573342

[B75] Shabbir U. Rubab M. Daliri E. B-M. Chelliah R. Javed A. Oh D-H. (2021). Curcumin, quercetin, catechins and metabolic diseases: The role of gut microbiota. Nutrients 13 (1), 206. 10.3390/nu13010206 33445760 PMC7828240

[B76] Sharma A. Kaur N. Sharma S. Rathore M. S. Ajay K. Mishra N. (2018). Embelin-loaded guar gum microparticles for the management of ulcerative colitis. J. Microencapsul. 35 (2), 181–191. 10.1080/02652048.2018.1452991 29544365

[B77] Shen C. Zhao L. Du X. Tian J. Yuan Y. Jia M. (2021). Smart responsive quercetin-conjugated glycol chitosan prodrug micelles for treatment of inflammatory bowel diseases. Mol. Pharm. 18 (3), 1419–1430. 10.1021/acs.molpharmaceut.0c01245 33522827

[B78] Shi J. Du P. Xie Q. Wang N. Li H. Smith E. E. (2020). Protective effects of tryptophan-catabolizing Lactobacillus plantarum KLDS 1.0386 against dextran sodium sulfate-induced colitis in mice. Food Funct. 11 (12), 10736–10747. 10.1039/d0fo02622k 33231244

[B79] Shin N. R. Whon T. W. Bae J. W. (2015). Proteobacteria: Microbial signature of dysbiosis in gut microbiota. Trends Biotechnol. 33 (9), 496–503. 10.1016/j.tibtech.2015.06.011 26210164

[B80] Siemer S. Hahlbrock A. Vallet C. McClements D. J. Balszuweit J. Voskuhl J. (2018). Nanosized food additives impact beneficial and pathogenic bacteria in the human gut: A simulated gastrointestinal study. Npj Sci. Food 2, 22. 10.1038/s41538-018-0030-8 30882042 PMC6420113

[B81] Skolmowska D. Głąbska D. Guzek D. Lech G. (2019). Association between dietary isoflavone intake and ulcerative colitis symptoms in polish caucasian individuals. Nutrients 11, 1936. 10.3390/nu11081936 31426486 PMC6722525

[B82] Sugimoto S. Naganuma M. Kanai T. (2016). Indole compounds may be promising medicines for ulcerative colitis. J. Gastroenterol. 51, 853–861. 10.1007/s00535-016-1220-2 27160749

[B83] Sun Q. Luan L. Arif M. Li J. Dong Q. J. Gao Y. (2018). Redox-sensitive nanoparticles based on 4-aminothiophenol- carboxymethyl inulin conjugate for budesonide delivery in inflammatory bowel diseases. Carbohydr. Polym. 189, 352–359. 10.1016/j.carbpol.2017.12.021 29580419

[B84] Suzuki T. Hara H. (2009). Quercetin enhances intestinal barrier function through the assembly of zonula [corrected] occludens-2, occludin, and claudin-1 and the expression of claudin-4 in Caco-2 cells. J. Nutri 139 (5), 965–974. 10.3945/jn.108.100867 19297429

[B85] Takagi T. Naito Y. Higashimura Y. Ushiroda C. Mizushima K. Ohashi Y. (2016). Partially hydrolysed guar gum ameliorates murine intestinal inflammation in association with modulating luminal microbiota and SCFA. Br. J. Nutr. 116 (7), 1199–1205. 10.1017/S0007114516003068 27604176

[B86] Takeuchi T. Miyauchi E. Kanaya T. Kato T. Nakanishi Y. Watanabe T. (2021). Acetate differentially regulates IgA reactivity to commensal bacteria. Nature 595, 560–564. 10.1038/s41586-021-03727-5 34262176

[B87] Tang H. Y. Fang Z. Ng K. (2020). Dietary fiber-based colon-targeted delivery systems for polyphenols. Trends Food Sci. Tech. 100, 333–348. 10.1016/j.tifs.2020.04.028

[B88] Tian L. Tan Y. Chen G. Wang G. Sun J. Ou S. (2019). Metabolism of anthocyanins and consequent effects on the gut microbiota. Crit. Rev. Food Sci. Nutr. 59, 982–991. 10.1080/10408398.2018.1533517 30595029

[B89] Tong L. Hao H. Zhang Z. Lv Y. Liang X. Liu Q. (2021). Milk-derived extracellular vesicles alleviate ulcerative colitis by regulating the gut immunity and reshaping the gut microbiota. Theranostics 11 (17), 8570–8586. 10.7150/thno.62046 34373759 PMC8344018

[B90] Valcheva R. Koleva P. Martínez I. Gänzle M. G. Walter J. Dieleman L. A. (2019). Inulin-type fructans improve active ulcerative colitis associated with microbiota changes and increased short-chain fatty acids levels. Gut Microbes 10 (3), 334–357. 10.1080/19490976.2018.1526583 30395776 PMC6546336

[B91] Varshosaz J. Minaiyan M. Khaleghi N. (2015). Eudragit nanoparticles loaded with silybin: A detailed study of preparation, freeze-drying condition and *in vitro*/*in vivo* evaluation. J. Microencapsul. 32 (3), 211–223. 10.3109/02652048.2014.995728 25561026

[B92] Wang Y. Tang Q. Duan P. Yang L. (2018). Curcumin as a therapeutic agent for blocking NF-κB activation in ulcerative colitis. Immunopharm Immunot 40 (6), 476–482. 10.1080/08923973.2018.1469145 30111198

[B93] Wu Z. Huang S. Li T. Han D. Zhang B. Xu Z. Z. (2021). Gut microbiota from green tea polyphenol-dosed mice improves intestinal epithelial homeostasis and ameliorates experimental colitis. Microbiome 9, 184. 10.1186/s40168-021-01115-9 34493333 PMC8424887

[B94] Xiong X. Cheng Z. Wu F. Hu M. Liu Z. Dong R. (2021). Berberine in the treatment of ulcerative colitis: A possible pathway through Tuft cells. Biomed. Pharmacother. 134, 111129. 10.1016/j.biopha.2020.111129 33348308

[B95] Xu Y. Zhu B. W. Li X. Li Y. F. Ye X. M. Hu J. N. (2022). Glycogen-based pH and redox sensitive nanoparticles with ginsenoside Rh2 for effective treatment of ulcerative colitis. Biomaterials 280, 121077. 10.1016/j.biomaterials.2021.121077 34890974

[B96] Xue J. Blesso C. Luo Y. (2023a). A comprehensive review of nanoparticles for oral delivery in food: Biological fate, evaluation models, and gut microbiota influences. Annu. Rev. Food Sci. Technol. 14, 1–33. 10.1146/annurev-food-060721-025159 36400014

[B97] Xue J. C. Yuan S. Meng H. Hou X. T. Li J. Zhang H. M. (2023b). The role and mechanism of flavonoid herbal natural products in ulcerative colitis. Biomed. Pharmacother. 158, 114086. 10.1016/j.biopha.2022.114086 36502751

[B98] Yang Z. H. Liu F. Zhu X. R. Suo F. Y. Jia Z. J. Yao S. K. (2021). Altered profiles of fecal bile acids correlate with gut microbiota and inflammatory responses in patients with ulcerative colitis. World J. Gastroenterol. 27, 3609–3629. 10.3748/wjg.v27.i24.3609 34239273 PMC8240054

[B99] Zakerska-Banaszak O. Tomczak H. Gabryel M. Baturo A. Wolko L. Michalak M. (2021). Dysbiosis of gut microbiota in polish patients with ulcerative colitis: A pilot study. Sci. Rep. 11 (1), 2166. 10.1038/s41598-021-81628-3 33495479 PMC7835370

[B100] Zam W. (2018). Gut microbiota as a prospective therapeutic target for curcumin: A review of mutual influence. J. Nutr. Metab. 2018, 1367984. 10.1155/2018/1367984 30647970 PMC6311836

[B101] Zhang M. Merlin D. (2018). Nanoparticle-based oral drug delivery systems targeting the colon for treatment of ulcerative colitis. Inflamm. Bowel Dis. 24 (7), 1401–1415. 10.1093/ibd/izy123 29788186 PMC6085987

[B102] Zhang M. Viennois E. Prasad M. Zhang Y. Wang L. Zhang Z. (2016). Edible ginger-derived nanoparticles: A novel therapeutic approach for the prevention and treatment of inflammatory bowel disease and colitis-associated cancer. Biomaterials 101, 321–340. 10.1016/j.biomaterials.2016.06.018 27318094 PMC4921206

[B103] Zhang M. Wang X. Han M. K. Collins J. F. Merlin D. (2017). Oral administration of ginger-derived nanolipids loaded with siRNA as a novel approach for efficient siRNA drug delivery to treat ulcerative colitis. Nanomedicine (Lond) 12 (16), 1927–1943. 10.2217/nnm-2017-0196 28665164 PMC5827822

[B104] Zhang Z. Peng X. Li S. Zhang N. Wang Y. Wei H. (2014). Isolation and identification of quercetin degrading bacteria from human fecal microbes. PLoS one 9, e90531. 10.1371/journal.pone.0090531 24594786 PMC3942438

[B105] Zheng L. X. Chen X. Q. Cheong K. L. (2020). Current trends in marine algae polysaccharides: The digestive tract, microbial catabolism, and prebiotic potential. Int. J. Biol. Macromol. 151, 344–354. 10.1016/j.ijbiomac.2020.02.168 32084473

[B106] Zhou Y. Xu Z. Z. He Y. Yang Y. Liu L. Lin Q. (2018). Gut microbiota offers universal biomarkers across ethnicity in inflammatory bowel disease diagnosis and infliximab response prediction. mSystems 3 (1), e00188-17. 10.1128/mSystems.00188-17 29404425 PMC5790872

[B107] Zhou Y. Zhi F. (2016). Lower level of Bacteroides in the gut microbiota is associated with inflammatory bowel disease: A meta-analysis. Biomed. Res. Int. 2016, 5828959. 10.1155/2016/5828959 27999802 PMC5143693

[B108] Zhu G. Wu X. Jiang S. Wang Y. Kong D. Zhao Y. (2022). The application of omics techniques to evaluate the effects of Tanshinone IIA on dextran sodium sulfate induced ulcerative colitis. Mol. Omics 18 (7), 666–676. 10.1039/d2mo00074a 35670211

[B109] Zou Q. Zhang X. Liu X. Li Y. Tan Q. Dan Q. (2020). Ficus carica polysaccharide attenuates DSS-induced ulcerative colitis in C57BL/6 mice. Food Funct. 11 (7), 6666–6679. 10.1039/d0fo01162b 32658237

[B110] Zu M. Ma Y. Cannup B. Xie D. Jung Y. Zhang J. (2021). Oral delivery of natural active small molecules by polymeric nanoparticles for the treatment of inflammatory bowel diseases. Adv. Drug Deliv. Rev. 176, 113887. 10.1016/j.addr.2021.113887 34314785

